# Reliability of rapid diagnostic test for diagnosing peripheral and placental malaria in an area of unstable malaria transmission in Eastern Sudan

**DOI:** 10.1186/1746-1596-8-59

**Published:** 2013-04-15

**Authors:** Awadalla H Kashif, Gamal K Adam, Ahmed A Mohmmed, Salah E Elzaki, Ahmed M AbdelHalim, Ishag Adam

**Affiliations:** 1 Faculty of Medical Laboratory Sciences, University of Khartoum, Khartoum, Sudan; 2 Faculty of Medicine, University of Gadarif, Gadarif, Sudan; 3 Faculty of Medicine, The National Ribat University, P.O. Box 1157, Khartoum, Sudan; 4 Tropical Medicine Research Institute, National Centre for Research, Khartoum, Sudan; 5 Faculty of Medicine, University of Khartoum, Khartoum, Sudan

**Keywords:** *Plasmodium falciparum*, Rapid diagnostic kit, Microscopy, Placental malaria, Pregnancy

## Abstract

**Background:**

Diagnosing *Plasmodium falciparum* malaria during pregnancy is a great challenge for clinicians because of the low density of parasites in the peripheral blood and parasite sequestration in the placenta. Nevertheless, few data on the use of malaria rapid diagnostic test (RDT) during pregnancy have been published.

**Methods:**

*P. falciparum* infections were assessed in 156 febrile pregnant women by microscopic examination of their blood smears and by RDT and polymerase chain reactions (PCR). In addition, 150 women were assessed at the time of delivery by microscopy, RDT, PCR and placental histology investigations. The study was conducted at the Gadarif Hospital, Eastern Sudan. The SD Bioline P. f / P. v (Bio Standard Diagnostics, Gurgaon, Korea) RDT kit was evaluated in this study.

**Results:**

Among the febrile pregnant women, 17 (11.0%), 26 (16.7%) and 18 (11.5%) positive cases of *P. falciparum* were detected by microscopy, RDT, and PCR, respectively. The sensitivity and specificity of the microscopy was 94.4% and 100%, respectively. The corresponding values for RDT evaluation were 83.3% and 92.0%, as compared with PCR as the gold standard.

While there were no detected cases of malaria by microscopic examination of blood smears, 27 (18.0%), 21(14.0%) and 46 (30.7%) out of the 150 placentae investigated had *P. falciparum* as determined by RDT, PCR, and histology, respectively. The sensitivity and specificity for RDT was 17.4% and 81.7%, respectively. The corresponding values for PCR were 6.5% and 82.7%, where histology was used as the gold standard.

**Conclusions:**

The RDT kit used in this study has poor performance for peripheral and placental *P. falciparum* malaria detection in this setting.

**Virtual slides:**

The virtual slide(s) for this article can be found here: http://www.diagnosticpathology.diagnomx.eu/vs/1092363465928479

## Background

Malaria is a major public health problem in the tropics. Around 125 million pregnant women live in malaria-endemic areas, and 32 million of these are at risk of malaria in sub-Saharan Africa [1,2]. Sequestration of malaria parasites in the placenta, where selection of pregnancy-associated *Plasmodium falciparum* erythrocyte membrane protein-1 (*Pf*EMP-1) variant surface antigen occurs in *P. falciparum* malaria in pregnancy, presents an enormous diagnostic challenge, especially in sub-Saharan African countries [3,4].

There is a need for accurate and prompt diagnosis of malaria so as to achieve the desired level of disease control. This goal is of fundamental importance because prescription of any drug during pregnancy poses a risk to the unborn child [5]. The World Health Organization (WHO) now recommends a parasite-based diagnosis of malaria infection [6]. Microscopic examination using Giemsa-stained capillary blood slides for detection of malaria parasites remains the reference standard [6]. However, blood film microscopy is time-consuming and needs significant technical skills, good-quality reagents, and clean slides; thus, its accuracy is of highly variable quality in sub-Saharan African hospitals [7-10]. Therefore, there is a need for the development of easier and faster diagnostic methods such as rapid diagnostic tests (RDTs). It has been shown that RDT is easy to use, are heat stable and have the ability to detect low parasitaemias [11]. Thus RDT is an ideal diagnostic tool for malaria diagnosis in settings that are resource constrained. While RDT have been extensively evaluated in malaria among the non-pregnant population, there are few published data on the performance of RDT for diagnosing malaria during pregnancy, especially in areas of unstable malaria transmission [12-17].

Pregnant Sudanese women are at risk of malaria regardless of their age or parity and malaria is associated with adverse maternal and perinatal outcomes [18]. RDT has not yet been implemented as standard methods for diagnosing malaria during pregnancy in Sudan. Hence, this study was conducted to investigate the performance of RDT in the diagnosis of *P. falciparum* infections in febrile pregnant women and placental malaria. This study aimed to provide evidence-based data on the best diagnostic methods for malaria during pregnancy, based on our recent observation of the limitations of the different diagnostic methods for malaria during pregnancy in Sudan [19-21].

## Methods

This study was conducted at Gadarif Hospital in Eastern Sudan during September through December 2011. The area is characterized by unstable malaria transmission and *P. falciparum* is the sole malaria species present [22]. The cross-sectional study at the antenatal clinic that recruited febrile (temperature ≥ 37.5°C) pregnant women, compared the accuracy of RDT with microscopy; PCR was used as a reference standard for detecting peripheral parasitaemia in the participants. At delivery, a second cross-sectional study compared the accuracy of RDT and microscopy with placental histology (the reference standard), and with PCR performed to detect placental malaria.

After signing informed consent documents, those enrolled in both studies (i.e., the pregnant and newly delivered women) were screened and clinically examined for malaria. Information was collected on social demographics using a pre-tested questionnaire. Malaria screening was performed using microscopy, RDT and PCR during pregnancy, and placental histology at delivery. Pregnant women with a confirmed diagnosis of malaria were treated with artesunate sulfadoxine-pyrimethamine which is the first line treatment for *P. falciparum* malaria in Sudan [23]. The sample sizes were calculated based on 2-sided hypothesis tests using Epi Info with 80% power and a confidence interval of 95%.

### Rapid diagnostic testing for malaria

The commercially available RDT kit SD Bioline P.f / P.v (Bio Standard Diagnostics, Gurgaon, Korea) was used in this study according to the manufacturer’s instructions. It is used for the qualitative detection of antigens produced by *P. falciparum* (Pf) and *P. vivax* (Pv). These antigens are the Histidine Rich Proteine-2 (PfHRP2) and lactate dehydrogenase (PvLD). Briefly a drop of whole blood (20 μl) was added to the card pad followed by three drops of lysis reagent. The RDT result was read within 10 minutes and the results recorded immediately. The tests were interpreted as follows: the presence of one coloured band (C control line) within the results window indicated a negative result, two coloured bands (test line 1 and C line) indicated *P. falciparum*, two coloured bands (test line 2 and C line) indicated *P. vivax,* while the presence of three coloured lines (test line 1 and 2 and C line) indicated a mixed infection with *P. falciparum* and *P. vivax*. Tests were considered valid when there was a coloured line in the control and invalid if the control (C) failed to appear in the results window.

### Microscopy

Blood smears were stained with 10% Giemsa and examined under the X100 oil immersion objective lens of a light microscope by two independent laboratory technologists that were blinded to each other's results. The number of asexual parasites was counted against 200 leucocytes, where an average leucocyte count of 8,000/μL was assumed. Before a smear was considered negative, 200 high power fields had been examined.

### Placental histology

Full thickness placental blocks (around 3 cm) were taken from each placenta and kept in neutral buffered formalin for histology. The buffer was used to prevent formalin pigment formation, which has similar optical characteristics and polarized light activity as malaria pigment [24]. Placental malaria infections were characterized as previously described by Bulmer *et al.*[25]; i.e., uninfected (no parasites or pigment), acute (parasites in intervillous spaces), chronic (parasites in maternal erythrocytes and pigment in fibrin, or cells within fibrin and/or chorionic villous syncytiotrophoblast or stroma), and past (no parasites and pigment confined to fibrin or cells within fibrin).

### Parasite DNA extraction and PCR

Parasite DNA extraction and PCR assays were performed as described in our recent work [19]. Briefly, three drops of blood were collected onto a piece of filter paper from maternal peripheral blood and the maternal side of the placenta for the first and second study, respectively. These samples were air-dried and stored at ambient temperature in individual sterile plastic bags. The specimens were transported for processing and analysis in the laboratory in Khartoum. Approximately 25 μl (around one third of a spot) of blood was punched out from the dried blood spots. The blood-impregnated filter paper piece was washed with distilled water and placed directly in a PCR tube containing 25 μl of all of the PCR reaction components. A negative control sample containing no template DNA and an internal positive control were used for quality control purposes. Genomic DNA was checked by using an assay based on a nested PCR for *P. falciparum* DNA [26].

### Quality control

All of the study research team were trained and had continuous supervision by the pathologist and site supervisor. RDT kits were purchased centrally and delivered to the hospital by the study team. The manufacturer's storage temperature specifications (4-30°C) were adhered to by monitoring the air temperature during transportation and storage. The slide and RDT results were read by two individuals blinded to each other's results. Placental histology smears were examined by a senior pathologist, blindly (AAM). Likewise, the PCR was performed by the research team (AH and SA) who were blind to the microscopy, RDT, and histology results.

### Ethics

The study received ethical clearance from the Research Board at the Faculty of Medical Laboratory Sciences, University of Khartoum, Sudan.

### Statistics

Data were analysed using SPSS (Statistical Package for the Social Sciences) software version 19.0. Sensitivity, specificity, positive and negative predictive values were determined as described previously [27].

## Results

The basic characteristics of the women enrolled in this study are shown in Table 1. The mean (SD) of the age, gestational age and temperature for the 156 febrile pregnant women was 27.0 (6.0) years, 19.0 (10.0) weeks and 38.2°C, respectively. Of these women, 17 (11.0%), 26 (16.7%) and 18 (11.5%) were *P. falciparum*-positive cases as detected by microscopy, RDT and PCR, respectively. The geometric mean of the parasite counts for the microscopy results was 8897.8 (1031.0) rings/μl.

**Table 1:** Mean (SD) basic characteristics of febrile pregnant women and parturient women at Gadarif Hospital, Eastern Sudan

**Variable**	**Pregnant febrile women (N= 156)**	**Parturient women (N = 150)**
Age, years	27.0(6.0)	26.0(6.0)
Parity	2.1(2.1)	1.6(1.7)
Gestational age, weeks	19.0(9.9)	38.8(3.6)
Temperature, °C	38.2(0.5)	37.4(0.5)
Weight, Kg	62.8(6.8)	63.1(7.5)
Haemoglobin, g/dl	10.3(1.3)	10.1(1.1)

**Figure 1:**
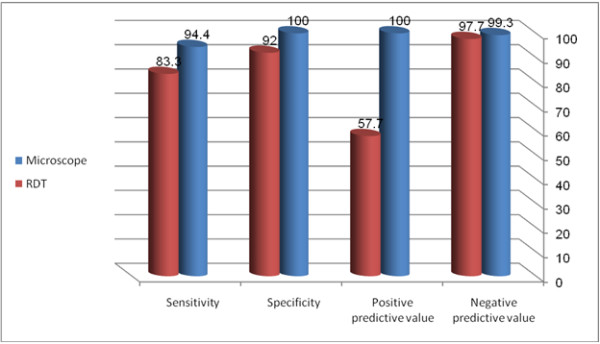
Accuracy of microscopy and RDT in comparison with PCR for detecting malaria infection among 156 febrile pregnant women in Eastern Sudan.

**Figure 2:**
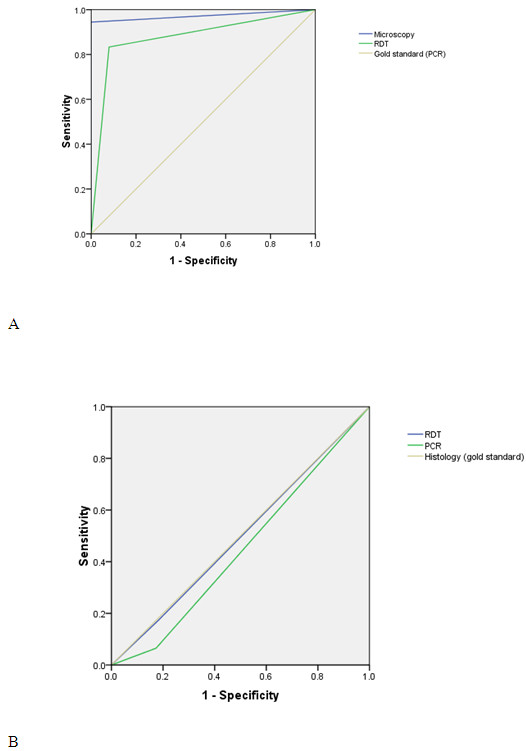
Comparison of receiver operator characteristic (ROC) curve for microscope RDT and PCR for diagnosing peripheral (A) and placental malaria (B).

The sensitivity, specificity, positive predictive value (PPV) and negative predictive value (NPV) for the microscopic analyses were 94.4%, 100%, 100% and 99.3%, respectively. The corresponding values for RDT were 83.3%, 92.0%, 57.7% and 97.7%, where PCR was used as the gold standard, Table 2, Figure 1 and Figure 2.

**Table 2:** Accuracy of microscopy and RDT in comparison with PCR for detecting malaria infection among 156 febrile pregnant women in Eastern Sudan

		**PCR**				
		**Positive**	**Negative**	**Total**	**Accuracy measure (95% CI)**	
**Microscopy**	Positive	17	0	17	Sensitivity	94.4 (75.5–99.7)
	Negative	1	138	139	Specificity	100 (98.0–100.0)
Total		18	138	156	Positive predictive value	100 (83.4–100.0)
					Negative predictive value	99.3 (96.5–100.0)
**RDT**	Positive	15	11	26	Sensitivity	83.3 (61.0–95.6)
	Negative	3	127	130	Specificity	92.0 (86.6–95.7)
Total		18	138	156	Positive predictive value	57.7 (38.4–75.4)
					Negative predictive value	97.7 (94.0–99.4)

While there were no detected cases of malaria in the microscopy examination, 27 (18.0%), 21 (14.0%) and 46 (30.7%) out of the 150 placentae investigated had *P. falciparum* infections as judged by RDT, PCR, and histology, respectively.

**Figure 3:**
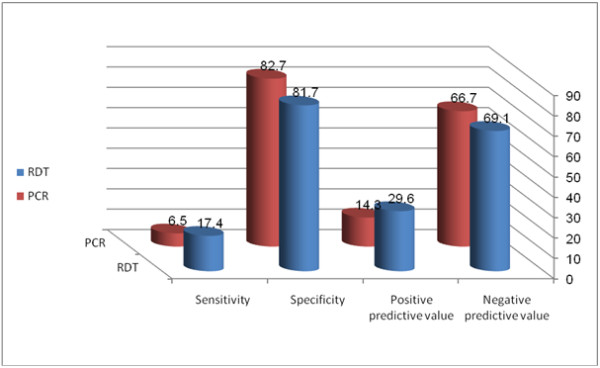
Accuracy of RDT and PCR in comparison with histology for detecting placental malaria infection at Gadarif Hospital, Eastern Sudan.

The sensitivity, specificity, PPV and NPV for the RDT kit were 17.4%, 81.7%, 29.6% and 69.6%, respectively. The corresponding values for PCR were 6.5%, 82.7%, 14.3% and 66.7%, where histology was used as the gold standard, Table 3, Figure 2 and Figure 3.

**Table 3:** Accuracy of RDT and PCR in comparison with histology for detecting placental malaria infection at Gadarif Hospital, Eastern Sudan

		**Histology**				
		**Positive**	**Negative**	**Total**	**Accuracy measure (95% CI)**	
**RDT**	Positive	8	19	27	Sensitivity	17.4(8.4–30.4)
	Negative	38	85	123	Specificity	81.7(73.4–88.3)
Total		46	104	150	Positive predictive value	29.6(14.8–48.6)
					Negative predictive value	69.1(60.5–67.8)
**PCR**	Positive	3	18	21	Sensitivity	6.5 (1.7–16.7)
	Negative	43	86	129	Specificity	82.7(74.5–89.0)
Total		46	104	150	Positive predictive value	14.3(3.8–34.1)
					Negative predictive value	66.7(58.2–74.4)

Likewise, the sensitivity, specificity, PPV and NPV for the RDT kit were low (14.3%, 81.4%, 11.1% and 85.43%, respectively), where PCR was used as the gold standard, Table 4.

**Table 4:** Accuracy of RDT in comparison with PCR for detecting placental malaria infection at Gadarif Hospital, Eastern Sudan

		**PCR**				
		**Positive**	**Negative**	**Total**	**Accuracy measure (95% CI)**	
**RDT**	Positive	3	24	27	Sensitivity	14.3 (3.8–34.1)
	Negative	18	105	123	Specificity	81.4 (74.0–87.4)
Total		21	129	150	Positive predictive value	11.1 (3.0–27.3)
					Negative predictive value	85.4 (78.3–90.8)

## Discussion

The main findings of the current study were that the RDT kit had moderate sensitivity (83.3%) and acceptable specificity (92.0%) for the diagnosis of peripheral *P. falciparum* among febrile pregnant women, but low sensitivity (17.4%) and specificity (81.7%) for diagnosing placental malaria when compared with PCR as the gold standard. The performance of the same brand of kit (SD Bioline P.f / P.v) was recently compared with PCR in eastern Sudan among febrile non-pregnant patients where it was found to have a sensitivity of 69% and specificity of 84% [28]. Recently low sensitivity (31.8%) but full specificity (100%) was reported for RDT kits used during pregnancy in Uganda, where PCR was used as the gold standard [13]. The performance of the RDT kit for malaria diagnosis used in the current study is in agreement with the findings of Schachterle *et al.*, who showed that RDT kits had high false positive and negative rates in a region of malaria hypoendemicity in Tanzania [29]. However, the results of the later study were based on microscopy data without PCR correction. Furthermore, Mayor et al., [16] have recently shown that among the 122 women that were PCR-positive for *P. falciparum* (as judged by peripheral and/or placental blood sampling) 87 (71.3%) and 74 (60.7%) were not considered positive by peripheral microscopy and the HRP2 RDT, respectively.

Nevertheless, it has been observed that RDT had high sensitivity (96.8%) and specificity (73.5%) for the diagnosis of *P. falciparum* malaria among febrile pregnant women in a hyper-endemic region in Uganda, when compared with microscopy as the gold standard [15]. However, it was observed that the RDT had a modest level of accuracy (80.9% sensitivity, 87.5% specificity) for detecting placental malaria using peripheral blood at time of delivery, in the later study [15]. Recently, the prevalence of placental infection, as determined by microscopy and RDT, was 5.1% and 5.0%, respectively, with highly significant agreement (82.9%); however discordances were observed between the two methods at low level parasitaemias [12]. Previous studies conducted at the time of birth have shown that RDT detecting *P. falciparum* HRP2 are more sensitive than blood smears, and appear to be reliable predictors of adverse outcomes of malaria in pregnancy [30-32].

Although the manufacturer’s (Bio Standard Diagnostics, Gurgaon, Korea) instructions were strictly followed, the poor performance this RDT kit in the current study is disappointing, and perhaps somewhat difficult to explain. Furthermore, the RDT used in the current study was very sensitive and specific when evaluated by WHO/ FIND [11]. High sensitivity is needed to provide confidence to the practicing physician that the RDT is unlikely to miss a malaria infection in pregnancy. However, the PPV was very low (14.3%); this could be due to false positive results, possibly attributable to the persistent nature of HRP-2 antigenaemia that has been documented already in previous studies [33,34]. It should be mentioned that HRP2 based RDT positivity among pregnant women can persist for up to 28 days after antimalarial drug treatment, especially among women with low gravidity and those with a higher parasite density at enrolment [14]. It seems to be still there is a great challenge in diagnosing malaria and its treatment adverse effects and associated anemia [35-37].

The Sudanese National Malaria Control Programme recommends the use of RDT in those settings where no expert microscopy is available, and maintains microscopic examination in those places where microscopy is of an adequate level. This RDT strategy was investigated earlier in Sudan for the home management of malaria using artemisinin-based combination therapy [38]. Therefore, based on the findings of the current study, it appears likely that implementation of malaria RDT in Sudan in settings where microscopic expertise is available should not be recommended.

## Conclusions

RDT has poor performance in detecting peripheral and placental *P. falciparum* malaria in this setting.

## Competing interests

The authors declare that they have no competing interests.

## Authors’ contributions

AHK and IA coordinated and carried out the study, and participated in the statistical analysis and procedures. GKA and AMA participated in the clinical work and statistical analyses. AAM and SEE conducted the laboratory work. All the authors have read and approved the final version of this manuscript.
